# Crystal structure and Hirshfeld surface analysis of 3-benzyl-2-[bis(1*H*-pyrrol-2-yl)methyl]thiophene

**DOI:** 10.1107/S2056989023010800

**Published:** 2024-01-01

**Authors:** Nurlana D. Sadikhova, Zeliha Atioğlu, Narmina A. Guliyeva, Evgeniya R. Shelukho, Darya K. Polyanskaya, Victor N. Khrustalev, Mehmet Akkurt, Ajaya Bhattarai

**Affiliations:** a Organic Chemistry Department, Baku State University, Az 1148 Baku, Azerbaijan; b Department of Aircraft Electrics and Electronics, School of Applied Sciences, Cappadocia University, Mustafapaşa, 50420 Ürgüp, Nevşehir, Türkiye; c Department of Organic Substances and Technology of High-Molecular Compounds, SRI "Geotechnological Problems of Oil, Gas and Chemistry", Azerbaijan State Oil and Industry University, Azadlig ave. 20, Az-1010 Baku, Azerbaijan; d RUDN University, 6 Miklukho-Maklaya St., Moscow 117198, Russian Federation; e Zelinsky Institute of Organic Chemistry of RAS, 4, 7 Leninsky Prospect, 119991 Moscow, Russian Federation; f Department of Physics, Faculty of Sciences, Erciyes University, 38039 Kayseri, Türkiye; g Department of Chemistry, M.M.A.M.C (Tribhuvan University) Biratnagar, Nepal; Texas A & M University, USA

**Keywords:** crystal structure, thio­phene ring, 1*H*-pyrrole ring, hydrogen bonds, Hirshfeld surface analysis

## Abstract

In the title compound, C_20_H_18_N_2_S, the asymmetric unit comprises two similar mol­ecules (*A* and *B*). In mol­ecule *A*, the central thio­phene ring makes dihedral angles of 89.96 (12) and 57.39 (13)° with the 1*H*-pyrrole rings, which are bent at 83.22 (14)° relative to each other, and makes an angle of 85.98 (11)° with the phenyl ring. In mol­ecule *B*, the corresponding dihedral angles are 89.49 (13), 54.64 (12)°, 83.62 (14)° and 85.67 (11)°, respectively. In the crystal, mol­ecular pairs are bonded to each other by N—H⋯N inter­actions. N—H⋯π and C—H⋯π inter­actions further connect the mol­ecules, forming a three-dimensional network. A Hirshfeld surface analysis indicates that H⋯H (57.1% for mol­ecule *A*; 57.3% for mol­ecule *B*), C⋯H/H⋯C (30.7% for mol­ecules *A* and *B*) and S⋯H/H⋯S (6.2% for mol­ecule *A*; 6.4% for mol­ecule *B*) inter­actions are the most important contributors to the crystal packing.

## Abstract 2

The asymmetric unit of the title compound contains two similar mol­ecules. In the crystal, mol­ecular pairs are bonded to each other by N—H⋯N inter­actions. N—H⋯π and C—H⋯π inter­actions further connect the mol­ecules, forming a three-dimensional network.

## 1. Chemical context

**Figure 1:**
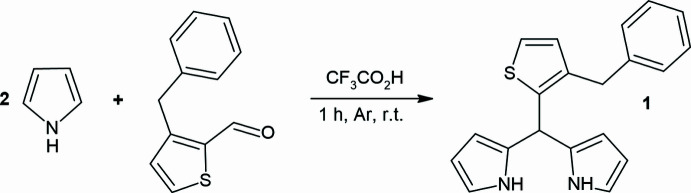
Synthesis of 3-benzyl-2-[bis(1*H*-pyrrol-2-yl)methyl]thiophene (**1**).

Dipyrro­methanes (Nascimento *et al.*, 2019 ▸) are well-known synthetic scaffolds for the synthesis of porphyrins (Lindsey, 2010 ▸; Yedukondalu, *et al.*, 2011 ▸), calixpyrroles (Gale *et al.*, 2001 ▸) and chlorins (Taniguchi *et al.*, 2017 ▸), corroles (Orłowski *et al.*, 2017 ▸). Other important uses of dipyrro­methanes include the synthesis of dipyrromethines and their complexes (Safavora *et al.*, 2019 ▸; Wood *et al.*, 2007 ▸), as fluorescent markers or in coordination compounds, including borondipyrromethenes, known as BODIPYs. The synthesis of dipyrro­methanes is generally based on the acid-catalyzed condensation of pyrrole with aldehydes or acyl­chlorides in an organic solvent. Despite the large number of examples of the synthesis of dipyrro­methanes, there is a lack of literature data on the synthesis of thio­phene-substituted dipyrro­methanes. Therefore, we used 3-benzyl­thio­phene­carboxaldehyde (Zaytsev *et al.*, 2023 ▸), which, when reacted with pyrrole, gives the target dipyrro­methane **1** in 70% yield (Fig. 1 ▸). On the other hand, attachment of a thio­phene or pyrrole moiety to the organic mol­ecules can lead to various sorts of inter­molecular non-covalent inter­actions, resulting in inter­esting coordination, catalytic supra­molecular, and solvatochromic properties (Gurbanov *et al.*, 2020*a* ▸,*b* ▸;Khalilov *et al.*, 2021 ▸; Mahmoudi *et al.*, 2017*a* ▸,*b* ▸; Mahmudov *et al.*, 2015 ▸). For example, attachment of a pyrrole moiety to ligands can create additional coord­ination sites and inter­esting supra­molecular architectures, which may affect their catalytic activity (Gurbanov *et al.*, 2022*a* ▸,*b* ▸; Ma *et al.*, 2017 ▸, 2021 ▸; Shikhaliyev *et al.*, 2019 ▸). 
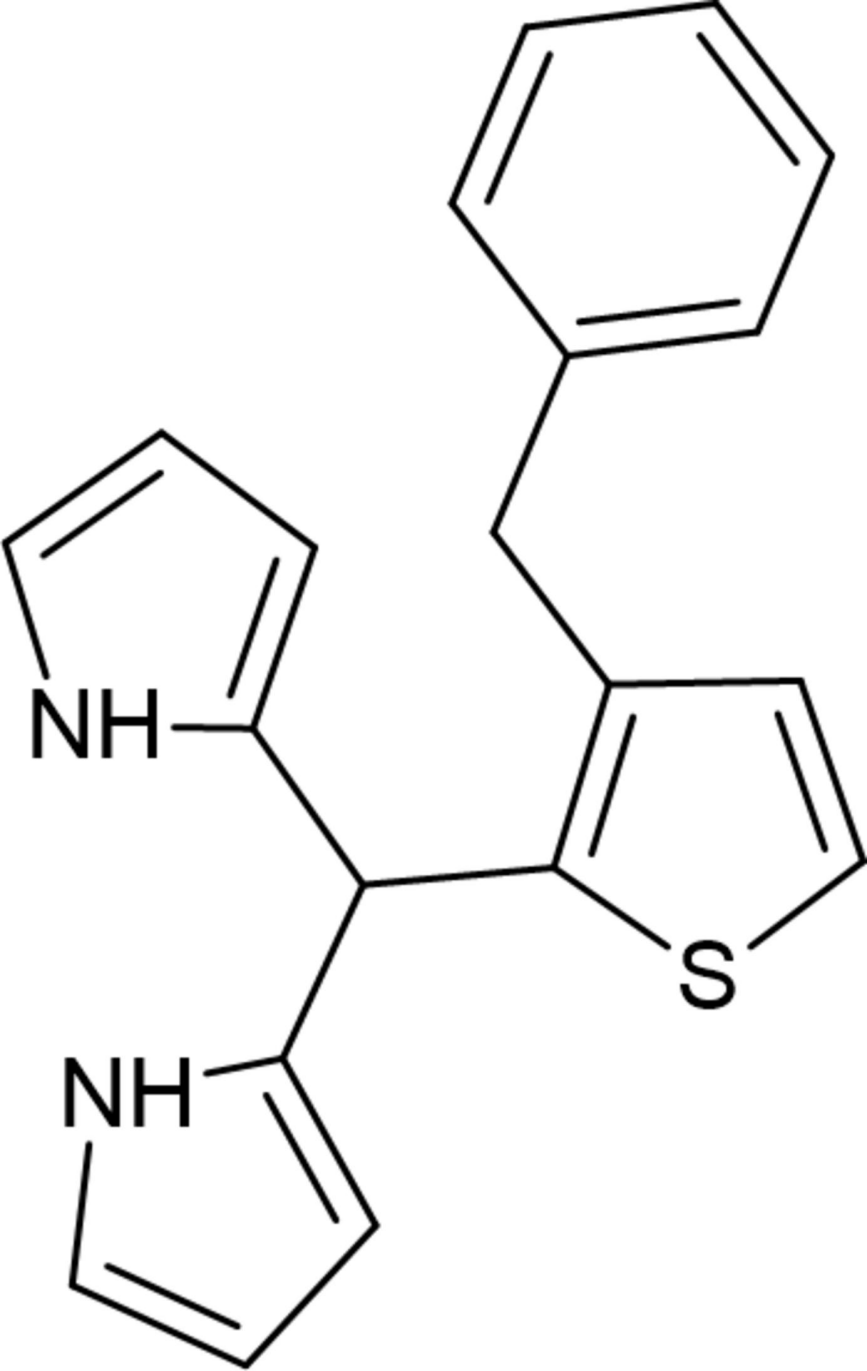


## 2. Structural commentary

**Figure 2:**
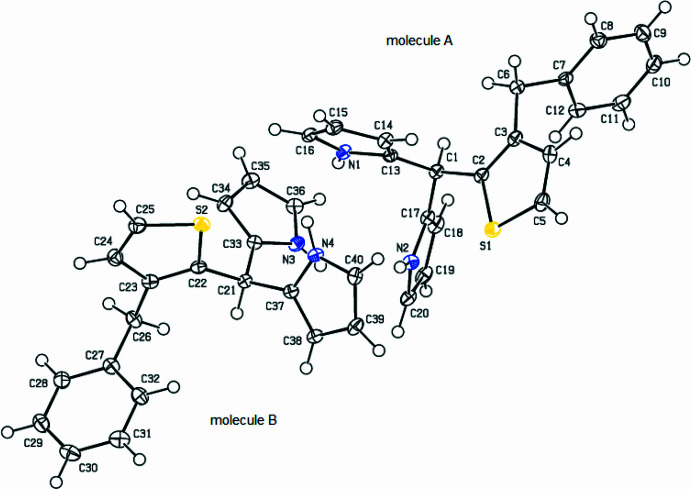
View of the two independent mol­ecules, *A* and *B*, in the asymmetric unit of the title compound, with displacement ellipsoids for the non-hydrogen atoms drawn at the 50% probability level.

**Figure 3:**
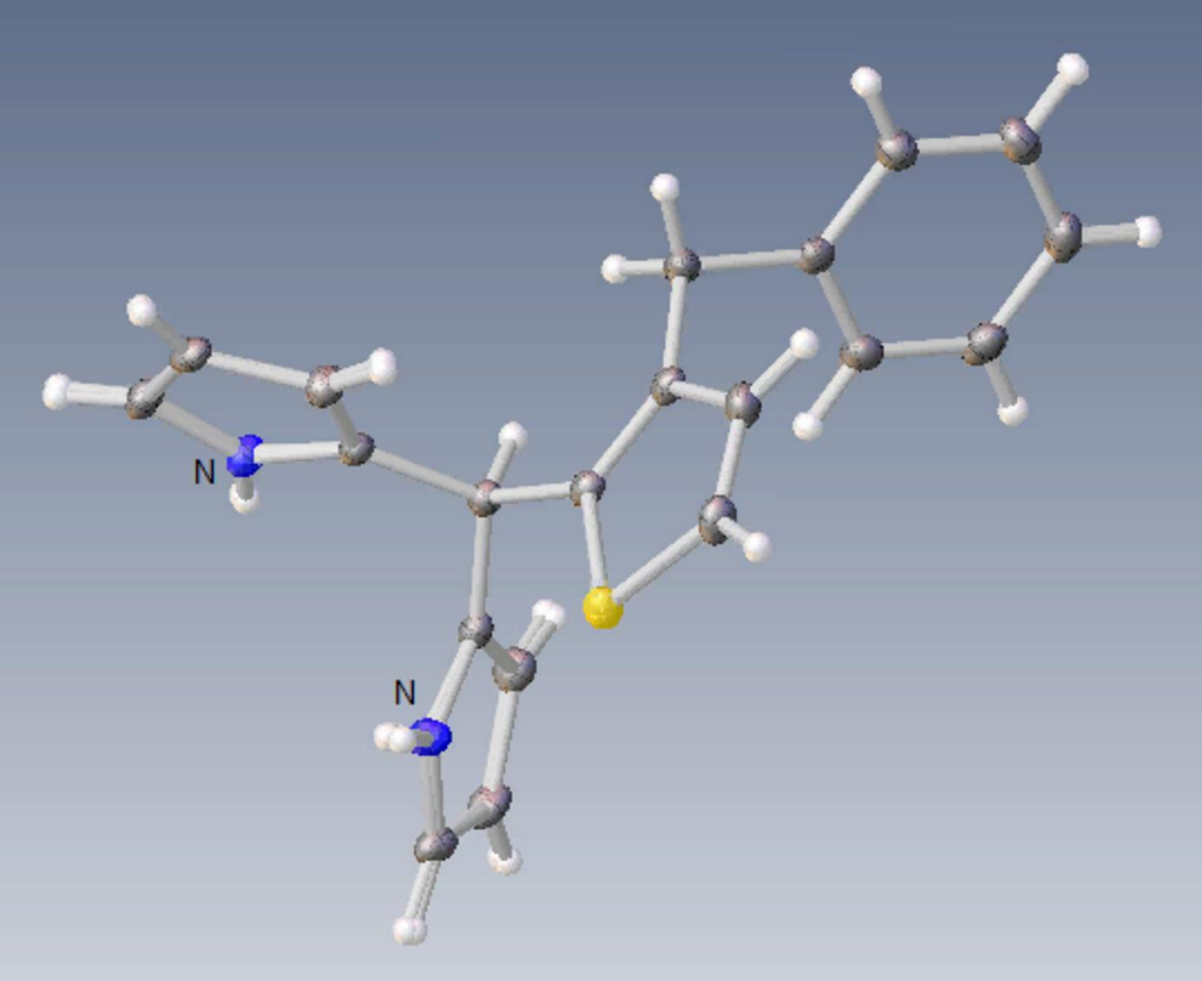
Overlay ball and stick image of the two independent mol­ecules (*A* and *B*) in the asymmetric unit of the title compound. Color code: carbon (gray), hydrogen (white), nitro­gen (blue) and sulfur (yellow).

As shown Fig. 2 ▸, the title compound crystallizes with two independent mol­ecules (*A* with the atom S1 and *B* with the atom S2) in the asymmetric unit. In mol­ecule *A*, the central thio­phene ring (S1/C2–C5) makes dihedral angles of 89.96 (12) and 57.39 (13)°, respectively, with the 1*H*-pyrrole rings (N1/C13–C16 and N2/C17–C20), which are bent at 83.22 (14)° relative to each other, and makes an angle of 85.98 (11)° with the phenyl ring (C7–C12). In mol­ecule *B*, the central thio­phene ring (S2/C22–C25) makes dihedral angles of 89.49 (13) and 54.64 (12)°, respectively, with the 1*H*-pyrrole rings (N3/C33–C36 and N4/C37–C40), which are bent at 83.62 (14)° relative to each other, and makes an angle of 85.67 (11)° with the phenyl ring (C27–C32). There is a weak inter­molecular N4—H4*N*⋯S2 inter­action (Table 1 ▸) in mol­ecule *B*. Fig. 3 ▸ shows the overlay of mol­ecules *A* and *B* in the asymmetric unit (r.m.s. deviation 0.055 Å). Bond lengths and angles in the mol­ecules of the title compound are comparable with those of closely related structures detailed in section 4 (*Database survey*).

## 3. Supra­molecular features and Hirshfeld surface analysis

In the crystal, mol­ecular pairs are bonded to each other by N1—H1*N*⋯N3 inter­actions (Tables 1 ▸ and 2 ▸). N—H⋯π and C–H⋯π inter­actions further connect the mol­ecules, forming a three-dimensional network (Table 1 ▸; Figs, 4 ▸, 5 ▸ and 6 ▸). π-π- stacking inter­actions are not observed.

**Figure 7:**
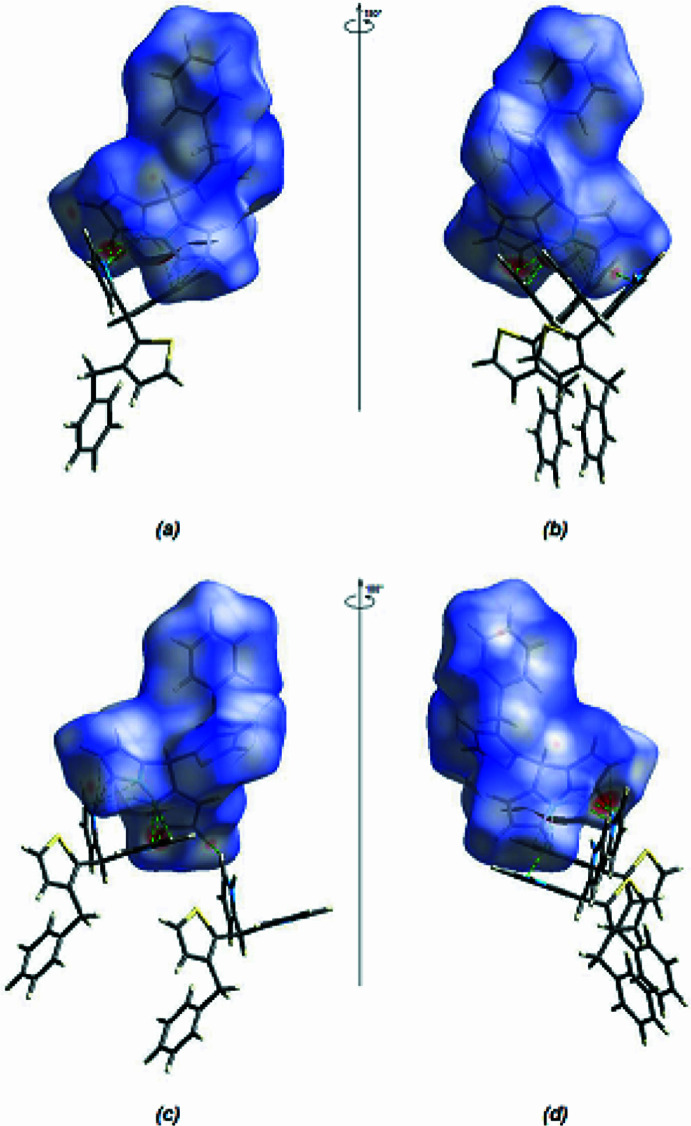
(*a*) Front and (*b*) back views for mol­ecule *A*, and (*c*) front and (*d*) back views for mol­ecule *B*, of the three-dimensional Hirshfeld surface for the title compound.

**Figure 8:**
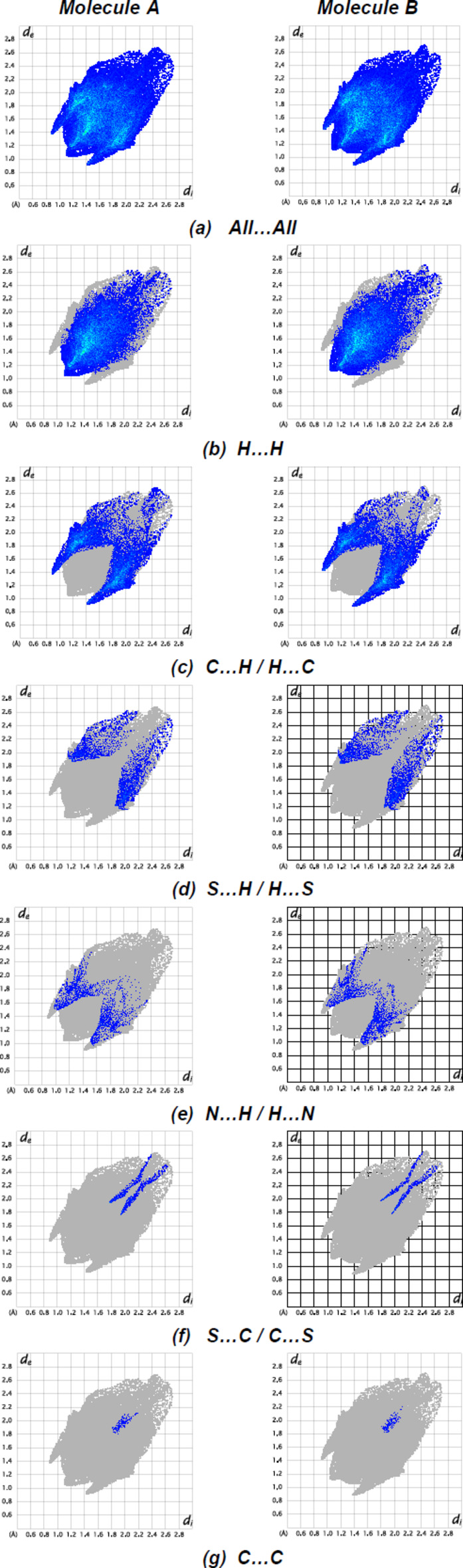
The two-dimensional fingerprint plots for the mol­ecules *A* and *B* of the title compound showing (*a*) all inter­actions, and delineated into (*b*) H⋯H, (*c*) C⋯H/H⋯C, (*d*) S⋯H/H⋯S, (*e*) N⋯H/H⋯N, (*f*) S⋯C/C⋯S and (*g*) C⋯C inter­actions. The *d*_i_ and *d*_e_ values are the closest inter­nal and external distances (in Å) from given points on the Hirshfeld surface.

*Crystal Explorer 17.5* (Spackman *et al.*, 2021 ▸) was used to generate Hirshfeld surfaces for both independent mol­ecules. The *d*_norm_ mappings for mol­ecules *A* and *B* were performed in the ranges −0.3807 to 1.3240 a.u. and −0.3811 to 1.3382 a.u., respectively. The N—H⋯N inter­actions are indicated by red areas on the Hirshfeld surfaces (Fig. 7 ▸ *a*,*b* for *A* and Fig. 7 ▸ *c*,*d* for *B*). Although H⋯H inter­actions (57.1% for mol­ecule *A* and 57.3% for mol­ecule *B*) contribute mainly to surface contacts, fingerprint plots (Fig. 8 ▸) show that C⋯H/H⋯C inter­actions (30.7% for mol­ecules *A* and *B*) are also significant (Tables 1 ▸ and 2 ▸). Other, less notable contacts are S⋯H/H⋯S (6.2% for mol­ecule *A* and 6.4% for mol­ecule *B*), N⋯H/H⋯N (4.0% contribution for mol­ecule *A* and 3.8% for mol­ecule *B*), S⋯C/C⋯S (1.5% for mol­ecule *A* and 1.3% for mol­ecule *B*) and C⋯C (0.4% for mol­ecules *A* and *B*). The comparison of the supplied data shows that mol­ecules *A* and *B* have extremely comparable environments.

## 4. Database survey

Three related compounds were found in a search of the Cambridge Structural Database (CSD, version 5.42, update of September 2021; Groom *et al.*, 2016 ▸), *viz*. 2-amino-*N*-(2-meth­oxy­phen­yl)-4,5-di­methyl­thio­phene-3-carboxamide (CSD refcode KODXEH; Chandra Kumar *et al.*, 2008 ▸), (2*E*)-1-(2,5-dimethyl-3-thien­yl)-3-(2-meth­oxy­phen­yl)prop-2-en-1-one (SUZQUA; Asiri *et al.*, 2010*a* ▸) and (*E*)-1-(2,5-dimethyl-3-thien­yl)-3-(2-hy­droxy­phen­yl)prop-2-en-1-one (SUYYUH; Asiri *et al.*, 2010*b* ▸). The crystal structure of KODXEH is consolidated by both inter- and intra­molecular N—H⋯O, C—H⋯O and C—H⋯N hydrogen bonds. In the crystal of SUZQUA, mol­ecules are linked by weak C—H⋯π and aromatic π–π stacking inter­actions [phenyl ring centroid–centroid separation = 3.6418 (11) Å; thio­phene– thio­phene ring separation = 3.8727 (9) Å]. In the crystal of SUYYUH, the mol­ecules are linked into polymeric chains extending along the *b*-axis direction by inter­molecular O—H⋯O hydrogen bonding. An *S*(6) ring motif (Bernstein *et al.*, 1995 ▸) is formed due to a short intra­molecular C—H⋯O contact. C—H⋯π inter­actions involving a methyl group of the 2,5-di­methyl­thienyl group and the benzene ring are present and π–π inter­actions between the centroids of the benzene and heterocyclic rings [3.7691 (9) Å] also occur.

## 5. Synthesis and crystallization

The starting 3-benzyl-2-thio­phencarboxaldehyde (0.38 g, 1.88 mmol) and pyrrole (3.15 g, 47 mmol) were placed into a two-neck flask. The reaction mixture was purged with argon for 10 min. Tri­fluoro­acetic acid (TFA, 21.4 mg, 0.19 mmol) was added dropwise to the reaction under stirring at r.t. After that, the reaction mixture was stirred for an hour under argon. Then Et_3_N (50 µL) was added to pH ∼7. The reaction mixture was poured into water (50 mL) and extracted with ethyl acetate (3 × 10 mL). The target product was purified by column chromatography (eluent: hepta­ne/ethyl acetate 10:1, TLC: hepta­ne/ethyl acetate 4:1). The title compound was obtained as a yellowish powder, which quickly darkened in air, yield 70%, 0.416 g (0.132 mmol); m.p. 390 K (with decomp.). A single crystal of the title compound was grown from a mixture of heptane and ethyl acetate (∼10:1). IR (KBr), *ν* (cm^−1^): *br*. 3413 (NH). ^1^H NMR (700.2 MHz, CDCl_3_) (*J*, Hz): *δ* 7.80 (*br.s*, 2H, NH), 7.27 (*t*, *J* = 7.6, 2H, H Ph), 7.20 (*t*, *J* = 7.6, 1H, H Ph), 7.13 (*d*, *J* = 5.0, 1H, H Thien), 7.08 (*d*, *J* = 7.6, 2H, H Ph), 6.82 (*d*, *J* = 5.0, 1H, H Thien), 6.69–6.68 (*m*, 2H, H Pyr), 6.08 (*dd*, *J* = 5.7, *J* = 2.6, 2H, H Pyr), 6.02-6.01 (*m*, 2H, H Pyr), 5.76 (*s*, 1H, CH), 3.91 (*s*, 2H, CH_2_). ^13^C{^1^H} NMR (176.1 MHz, CDCl_3_): *δ* 140.5, 140.3, 137.0, 131.7, 129.8 (2C), 128.6 (2C), 128.5 (2C), 126.2, 123.3, 117.3 (2C), 108.5 (2C), 107.2 (2C), 37.1, 34.3. GCMS (EI, 70 eV) *m/z* (%): [*M*]^+^ 318 (100), 250 (63), 239 (33), 227 (16), 184 (11), 174 (45), 91 (12). Elemental analysis calculated (%) for C_20_H_18_N_2_S: C 75.44, H 5.70, N 8.80, S 10.07; found: C 75.67, H 5.41, N 9.09, S 9.81.

## 6. Refinement

Crystal data, data collection and structure refinement details are summarized in Table 3 ▸. C-bound H atoms were included in the refinement using the riding-model approximation with C—H distances of 0.95–0.99 Å, and with *U*_iso_(H) = 1.2 or 1.5*U*_eq_(C). The H atoms of the NH groups were found from a difference map and refined with *U*_iso_(H) = 1.2*U*_eq_(N).

## Supplementary Material

Crystal structure: contains datablock(s) I. DOI: 10.1107/S2056989023010800/jy2043sup1.cif

Structure factors: contains datablock(s) I. DOI: 10.1107/S2056989023010800/jy2043Isup2.hkl

CCDC reference: 2319528

Additional supporting information:  crystallographic information; 3D view; checkCIF report

## Figures and Tables

**Figure 4:**
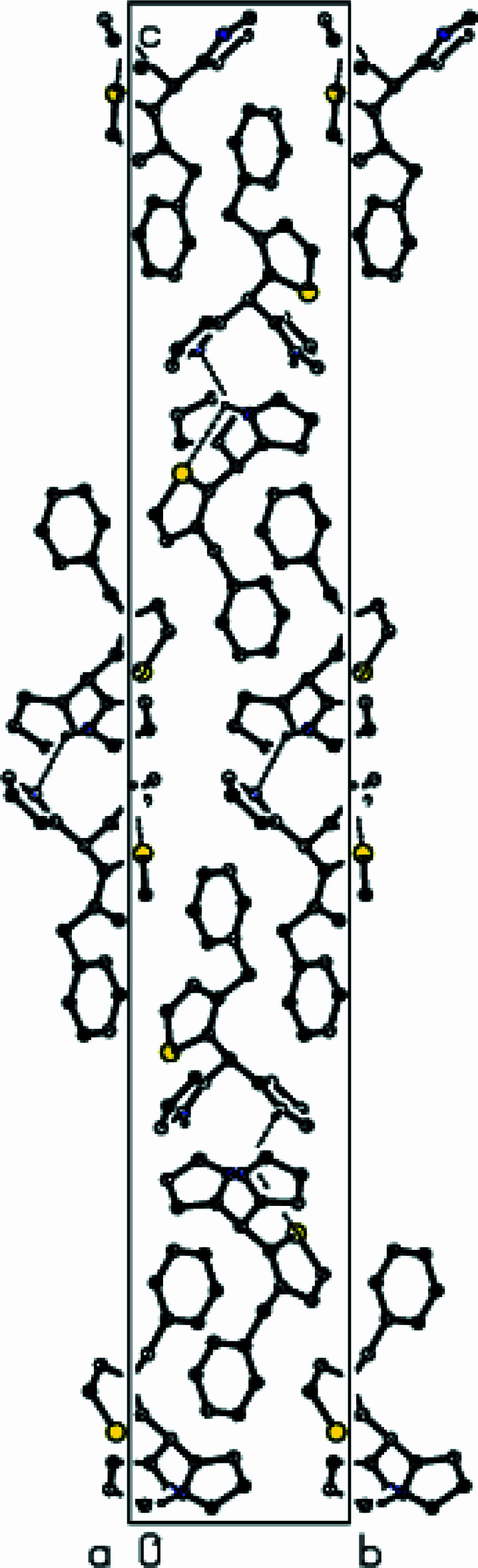
Packing of mol­ecules in the title compound with the N—H⋯N and N—H⋯S hydrogen bonds, viewed along the *a* axis.

**Figure 5:**
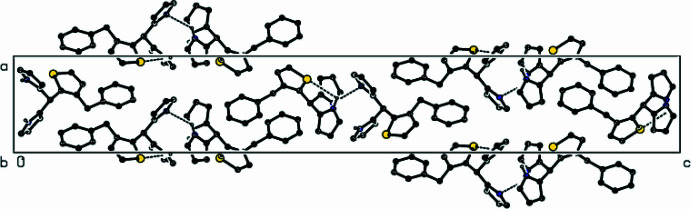
Packing of mol­ecules in the title compound, viewed along the *b* axis.

**Figure 6:**
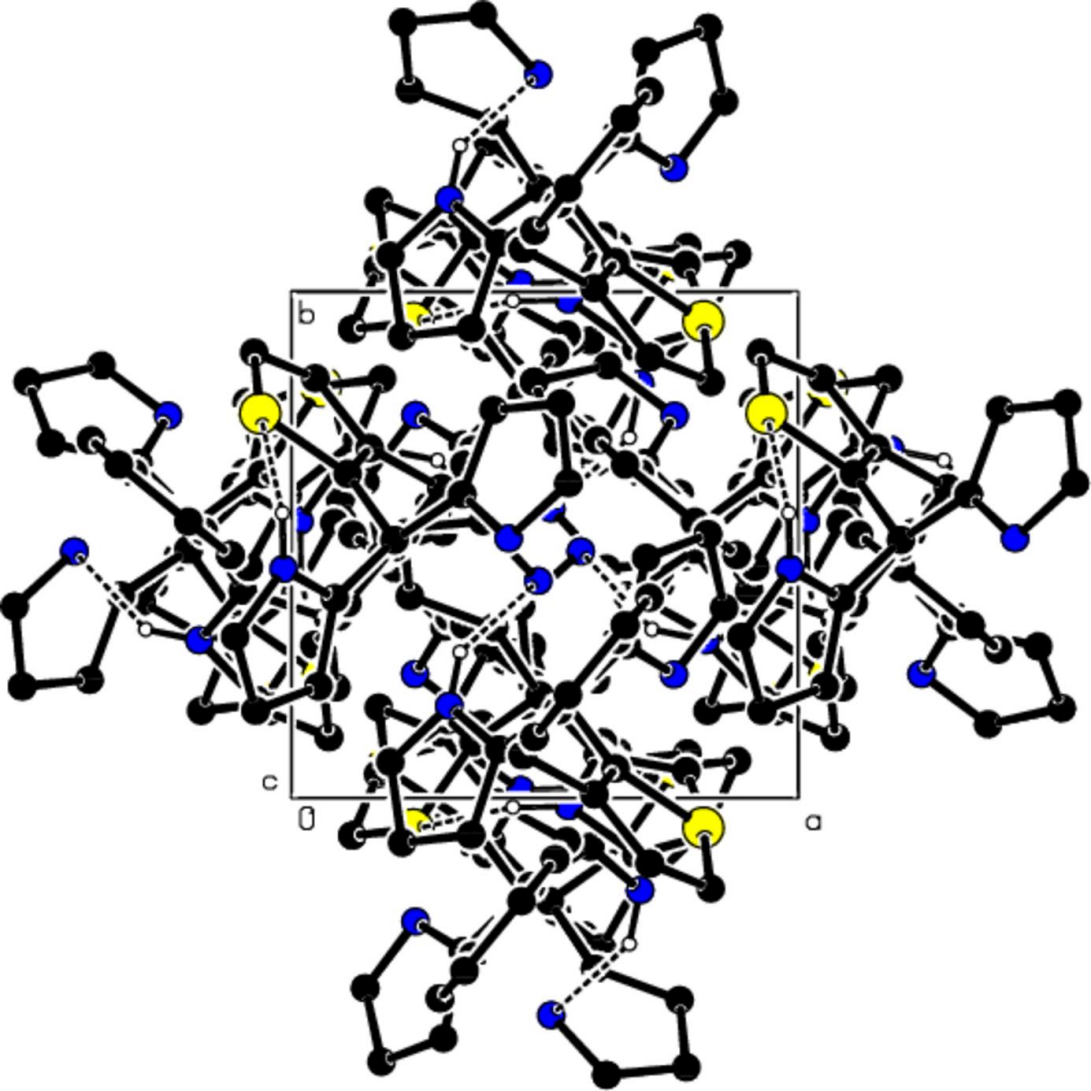
Packing of mol­ecules in the title compound, viewed along the *c* axis, with inter­actions depicted as in Fig. 4 ▸.

**Table 1:** Hydrogen-bond geometry (Å, °) *Cg*1–8 are the centroids of the S1/C2–C5, N1/C13–C16, N2/C17–C20, C7–C12, S2/C22–C25, N3/C33–C36, N4/C37–C40 and C27–C32 rings, respectively.

*D*—H⋯*A*	*D*—H	H⋯*A*	*D*⋯*A*	*D*—H⋯*A*
N1—H1*N*⋯N3	0.87 (3)	2.61 (3)	3.270 (3)	134 (3)
N4—H4*N*⋯S2	0.91 (3)	2.86 (3)	3.191 (2)	103 (2)
N1—H1*N*⋯*Cg*6	0.87 (3)	2.65 (3)	3.300 (2)	133 (3)
N2—H2*N*⋯*Cg*7	0.84 (3)	2.53 (3)	3.249 (2)	145 (3)
N3—H3*N*⋯*Cg*3	0.87 (3)	2.70 (3)	3.335 (2)	131 (3)
N4—H4*N*⋯*Cg*2	0.91 (3)	2.51 (3)	3.207 (2)	134 (3)
C5—H5⋯*Cg*8^i^	0.95	2.98	3.931 (3)	177
C6—H6*B*⋯*Cg*8^ii^	0.99	2.79	3.697 (3)	153
C10—H10⋯*Cg*7^iii^	0.95	2.86	3.544 (3)	130
C11—H11⋯*Cg*5^iii^	0.95	2.98	3.874 (3)	157
C25—H25⋯*Cg*4^iv^	0.95	2.98	3.924 (3)	176
C26—H26*A*⋯*Cg*4^v^	0.99	2.77	3.684 (3)	153
C30—H30⋯*Cg*3^vi^	0.95	2.88	3.585 (3)	132
C31—H31⋯*Cg*1^vi^	0.95	2.97	3.863 (3)	156

Symmetry codes: (i) 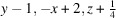 ; (ii) 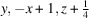 ; (iii) 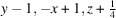 ; (iv) 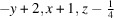 ; (v) 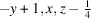 ; (vi) 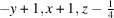 .

**Table 2:** Summary of short inter­atomic contacts (Å) in the title compound

Contact	Distance	Symmetry operation
C5⋯H18	3.01	*x*, 1 + *y*, *z*
H10⋯C40	3.01	−1 + *y*, 1 − *x*, 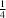 + *z*
H2*N*⋯C40	2.42	*x*, *y*, *z*
H4⋯C25	2.97	−1 + *y*, 2 − *x*, 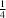 + *z*
H1⋯C29	2.79	*y*, 1 − *x*, 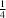 + *z*
H15⋯H35	2.53	*x*, 1 + *y*, *z*
H16⋯H20	2.51	1 + *x*, *y*, *z*
H16⋯H39	2.42	1 + *x*, *y*, *z*
H19⋯H40	2.30	1 *x*, −1 + *y*, *z*
H6*A*⋯H26*B*	2.43	*y*, 2 − *x*, 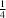 + *z*
H15⋯H19	2.59	1 + *x*, 1 + *y*, *z*
C25⋯H38	3.02	1 + *x*, *y*, *z*
H35⋯H20	2.42	1 + *x*, *y*, *z*
H36⋯H40	2.51	*x*, −1 + *y*, *z*

**Table 3:** Experimental details

Crystal data	
Chemical formula	C_20_H_18_N_2_S
*M*_r_	318.42
Crystal system, space group	Tetragonal, *P*4_3_
Temperature (K)	100
*a*, *c* (Å)	7.74413 (3), 53.4131 (3)
*V* (Å^3^)	3203.27 (3)
*Z*	8
Radiation type	Cu *K*α
μ (mm^−1^)	1.78
Crystal size (mm)	0.18 × 0.15 × 0.12

Data collection	
Diffractometer	Rigaku XtaLAB Synergy-S, HyPix-6000HE area-detector
Absorption correction	Multi-scan (*CrysAlis PRO*; Rigaku OD, 2021 ▸)
*T*_min_, *T*_max_	0.724, 1.000
No. of measured, independent and observed [*I* > 2σ(*I*)] reflections	25893, 5429, 5366
*R*_int_	0.034
(sin θ/λ)_max_ (Å^−1^)	0.639

Refinement	
*R*[*F* ^2^ > 2σ(*F* ^2^)], *wR*(*F* ^2^), *S*	0.029, 0.077, 1.03
No. of reflections	5429
No. of parameters	428
No. of restraints	1
H-atom treatment	H atoms treated by a mixture of independent and constrained refinement
Δρ_max_, Δρ_min_ (e Å^−3^)	0.32, −0.23
Absolute structure	Flack *x* determined using 1866 quotients [(*I* ^+^)-(*I* ^-^)]/[(*I* ^+^)+(*I* ^-^)] (Parsons *et al.*, 2013 ▸)
Absolute structure parameter	0.003 (10)

Computer programs: *CrysAlis PRO* (Rigaku OD, 2021 ▸), *SHELXT2016/6* (Sheldrick, 2015*a* ▸), *SHELXL2016/6* (Sheldrick, 2015*b* ▸), *ORTEP-3 for Windows* (Farrugia, 2012 ▸) and *PLATON* (Spek, 2020 ▸).

## supplementary crystallographic information

### Crystal data

**Table:**

C_20_H_18_N_2_S	*D*_x_ = 1.321 Mg m^−^^3^
*M**_r_* = 318.42	Cu *K*α radiation, λ = 1.54184 Å
Tetragonal, *P*4_3_	Cell parameters from 21419 reflections
*a* = 7.74413 (3) Å	θ = 3.3–79.7°
*c* = 53.4131 (3) Å	µ = 1.78 mm^−^^1^
*V* = 3203.27 (3) Å^3^	*T* = 100 K
*Z* = 8	Prism, yellow
*F*(000) = 1344	0.18 × 0.15 × 0.12 mm

### Data collection

**Table:**

Rigaku XtaLAB Synergy-S, HyPix-6000HE area-detector diffractometer	5366 reflections with *I* > 2σ(*I*)
Radiation source: micro-focus sealed X-ray tube	*R*_int_ = 0.034
φ and ω scans	θ_max_ = 80.0°, θ_min_ = 3.3°
Absorption correction: multi-scan (CrysAlisPro; Rigaku OD, 2021)	*h* = −9→9
*T*_min_ = 0.724, *T*_max_ = 1.000	*k* = −9→9
25893 measured reflections	*l* = −48→67
5429 independent reflections	

### Refinement

**Table:**

Refinement on *F*^2^	Hydrogen site location: mixed
Least-squares matrix: full	H atoms treated by a mixture of independent and constrained refinement
*R*[*F*^2^ > 2σ(*F*^2^)] = 0.029	*w* = 1/[σ^2^(*F*_o_^2^) + (0.0493*P*)^2^ + 0.7104*P*] where *P* = (*F*_o_^2^ + 2*F*_c_^2^)/3
*wR*(*F*^2^) = 0.077	(Δ/σ)_max_ = 0.001
*S* = 1.03	Δρ_max_ = 0.32 e Å^−^^3^
5429 reflections	Δρ_min_ = −0.23 e Å^−^^3^
428 parameters	Extinction correction: SHELXL, Fc^*^=kFc[1+0.001xFc^2^λ^3^/sin(2θ)]^-1/4^
1 restraint	Extinction coefficient: 0.00026 (3)
Primary atom site location: difference Fourier map	Absolute structure: Flack *x* determined using 1866 quotients [(*I*^+^)-(*I*^-^)]/[(*I*^+^)+(*I*^-^)] (Parsons *et al.*, 2013)
Secondary atom site location: difference Fourier map	Absolute structure parameter: 0.003 (10)

### Special details

**Table:**

Geometry. All esds (except the esd in the dihedral angle between two l.s. planes) are estimated using the full covariance matrix. The cell esds are taken into account individually in the estimation of esds in distances, angles and torsion angles; correlations between esds in cell parameters are only used when they are defined by crystal symmetry. An approximate (isotropic) treatment of cell esds is used for estimating esds involving l.s. planes.

### Fractional atomic coordinates and isotropic or equivalent isotropic displacement parameters (Å^2^)

**Table:**

	*x*	*y*	*z*	*U*_iso_*/*U*_eq_	
S1	0.18679 (6)	1.05845 (6)	0.55945 (2)	0.01754 (12)	
N1	0.6884 (2)	0.8179 (2)	0.52180 (4)	0.0144 (3)	
H1N	0.667 (4)	0.712 (4)	0.5175 (6)	0.017*	
N2	0.2448 (2)	0.7561 (2)	0.51944 (4)	0.0156 (3)	
H2N	0.253 (4)	0.854 (4)	0.5127 (6)	0.019*	
C1	0.4553 (3)	0.8123 (2)	0.55478 (4)	0.0142 (4)	
H1	0.5195	0.7343	0.5664	0.017*	
C2	0.3604 (3)	0.9414 (3)	0.57107 (4)	0.0145 (4)	
C3	0.4018 (3)	0.9926 (3)	0.59474 (4)	0.0161 (4)	
C4	0.2925 (3)	1.1294 (3)	0.60345 (4)	0.0191 (4)	
H4	0.3031	1.1805	0.6195	0.023*	
C5	0.1722 (3)	1.1786 (3)	0.58638 (5)	0.0198 (4)	
H5	0.0903	1.2681	0.5890	0.024*	
C6	0.5431 (3)	0.9154 (3)	0.61070 (4)	0.0176 (4)	
H6A	0.6237	1.0080	0.6159	0.021*	
H6B	0.6088	0.8307	0.6006	0.021*	
C7	0.4729 (3)	0.8264 (3)	0.63390 (5)	0.0159 (4)	
C8	0.5202 (3)	0.8793 (3)	0.65777 (5)	0.0194 (4)	
H8	0.5985	0.9728	0.6597	0.023*	
C9	0.4541 (3)	0.7964 (3)	0.67890 (5)	0.0224 (5)	
H9	0.4865	0.8348	0.6951	0.027*	
C10	0.3415 (3)	0.6587 (3)	0.67638 (5)	0.0224 (5)	
H10	0.2976	0.6018	0.6908	0.027*	
C11	0.2933 (3)	0.6044 (3)	0.65259 (5)	0.0225 (5)	
H11	0.2159	0.5101	0.6507	0.027*	
C12	0.3579 (3)	0.6878 (3)	0.63148 (5)	0.0204 (4)	
H12	0.3238	0.6502	0.6153	0.024*	
C13	0.5903 (2)	0.9037 (2)	0.53919 (4)	0.0134 (4)	
C14	0.6455 (3)	1.0726 (3)	0.53961 (4)	0.0164 (4)	
H14	0.6009	1.1623	0.5499	0.020*	
C15	0.7816 (3)	1.0884 (3)	0.52183 (5)	0.0173 (4)	
H15	0.8446	1.1904	0.5180	0.021*	
C16	0.8047 (3)	0.9288 (3)	0.51117 (4)	0.0162 (4)	
H16	0.8870	0.9004	0.4986	0.019*	
C17	0.3384 (2)	0.6979 (3)	0.53944 (4)	0.0144 (4)	
C18	0.2996 (3)	0.5255 (3)	0.54269 (5)	0.0180 (4)	
H18	0.3461	0.4513	0.5552	0.022*	
C19	0.1777 (3)	0.4791 (3)	0.52405 (5)	0.0195 (5)	
H19	0.1273	0.3683	0.5217	0.023*	
C20	0.1457 (3)	0.6246 (3)	0.50987 (5)	0.0178 (4)	
H20	0.0692	0.6324	0.4960	0.021*	
S2	0.75956 (6)	1.06184 (6)	0.44014 (2)	0.01709 (12)	
N3	0.5130 (2)	0.5712 (2)	0.47912 (4)	0.0155 (3)	
H3N	0.406 (4)	0.596 (4)	0.4827 (6)	0.019*	
N4	0.4526 (2)	1.0157 (2)	0.47943 (4)	0.0146 (3)	
H4N	0.563 (4)	1.018 (4)	0.4851 (6)	0.018*	
C21	0.5097 (2)	0.7967 (2)	0.44507 (4)	0.0133 (4)	
H21	0.4324	0.7315	0.4334	0.016*	
C22	0.6404 (3)	0.8894 (2)	0.42877 (4)	0.0143 (4)	
C23	0.6913 (3)	0.8456 (3)	0.40510 (4)	0.0155 (4)	
C24	0.8293 (3)	0.9530 (3)	0.39620 (5)	0.0189 (4)	
H24	0.8801	0.9410	0.3801	0.023*	
C25	0.8798 (3)	1.0738 (3)	0.41317 (5)	0.0195 (4)	
H25	0.9702	1.1545	0.4104	0.023*	
C26	0.6123 (3)	0.7043 (3)	0.38929 (4)	0.0176 (4)	
H26A	0.5272	0.6397	0.3995	0.021*	
H26B	0.7039	0.6226	0.3841	0.021*	
C27	0.5235 (3)	0.7752 (3)	0.36613 (4)	0.0167 (4)	
C28	0.5778 (3)	0.7287 (3)	0.34221 (5)	0.0197 (4)	
H28	0.6717	0.6510	0.3403	0.024*	
C29	0.4953 (3)	0.7956 (3)	0.32102 (5)	0.0224 (5)	
H29	0.5339	0.7635	0.3048	0.027*	
C30	0.3575 (3)	0.9084 (3)	0.32360 (5)	0.0221 (5)	
H30	0.3011	0.9534	0.3092	0.027*	
C31	0.3025 (3)	0.9549 (3)	0.34739 (5)	0.0218 (5)	
H31	0.2078	1.0318	0.3493	0.026*	
C32	0.3852 (3)	0.8897 (3)	0.36853 (5)	0.0195 (4)	
H32	0.3472	0.9234	0.3847	0.023*	
C33	0.5996 (3)	0.6628 (3)	0.46113 (4)	0.0141 (4)	
C34	0.7663 (3)	0.6010 (3)	0.46030 (5)	0.0171 (4)	
H34	0.8557	0.6398	0.4495	0.021*	
C35	0.7796 (3)	0.4683 (3)	0.47860 (5)	0.0177 (4)	
H35	0.8798	0.4021	0.4823	0.021*	
C36	0.6219 (3)	0.4531 (3)	0.48999 (4)	0.0163 (4)	
H36	0.5931	0.3750	0.5030	0.020*	
C37	0.3946 (3)	0.9145 (2)	0.46007 (4)	0.0140 (4)	
C38	0.2205 (3)	0.9467 (3)	0.45723 (4)	0.0162 (4)	
H38	0.1461	0.8951	0.4452	0.019*	
C39	0.1728 (3)	1.0709 (3)	0.47547 (5)	0.0178 (4)	
H39	0.0606	1.1174	0.4780	0.021*	
C40	0.3186 (3)	1.1115 (3)	0.48886 (4)	0.0167 (4)	
H40	0.3254	1.1917	0.5023	0.020*	

### Atomic displacement parameters (Å^2^)

**Table:**

	*U*^11^	*U*^22^	*U*^33^	*U*^12^	*U*^13^	*U*^23^
S1	0.0165 (2)	0.0187 (2)	0.0175 (3)	0.00554 (16)	0.00010 (19)	0.00311 (19)
N1	0.0122 (7)	0.0127 (8)	0.0183 (9)	−0.0003 (6)	0.0008 (7)	−0.0008 (7)
N2	0.0155 (8)	0.0125 (8)	0.0189 (9)	−0.0009 (6)	−0.0012 (7)	0.0011 (7)
C1	0.0134 (8)	0.0126 (8)	0.0165 (11)	0.0008 (7)	0.0005 (8)	0.0017 (8)
C2	0.0118 (8)	0.0141 (9)	0.0177 (10)	0.0003 (7)	0.0016 (8)	0.0030 (8)
C3	0.0127 (8)	0.0169 (9)	0.0188 (11)	−0.0003 (7)	0.0027 (8)	0.0018 (8)
C4	0.0197 (9)	0.0198 (10)	0.0177 (11)	0.0013 (8)	0.0047 (8)	−0.0011 (8)
C5	0.0184 (10)	0.0180 (10)	0.0230 (12)	0.0052 (7)	0.0054 (9)	0.0022 (8)
C6	0.0144 (9)	0.0226 (10)	0.0158 (10)	0.0002 (7)	0.0000 (8)	0.0011 (8)
C7	0.0115 (8)	0.0185 (9)	0.0177 (10)	0.0041 (7)	−0.0007 (7)	0.0020 (8)
C8	0.0190 (10)	0.0200 (10)	0.0192 (11)	0.0019 (8)	−0.0014 (8)	−0.0014 (8)
C9	0.0243 (11)	0.0265 (11)	0.0165 (12)	0.0062 (9)	−0.0007 (9)	0.0003 (9)
C10	0.0171 (10)	0.0274 (11)	0.0227 (12)	0.0057 (8)	0.0048 (9)	0.0062 (9)
C11	0.0149 (9)	0.0246 (11)	0.0281 (13)	0.0004 (8)	−0.0006 (9)	0.0045 (9)
C12	0.0160 (9)	0.0248 (10)	0.0203 (11)	0.0012 (8)	−0.0029 (8)	−0.0003 (9)
C13	0.0112 (8)	0.0142 (9)	0.0148 (10)	0.0012 (7)	−0.0013 (7)	−0.0003 (7)
C14	0.0155 (9)	0.0139 (9)	0.0198 (11)	−0.0003 (7)	0.0015 (8)	−0.0006 (8)
C15	0.0137 (9)	0.0167 (9)	0.0216 (12)	−0.0026 (7)	−0.0006 (8)	0.0028 (8)
C16	0.0118 (9)	0.0182 (9)	0.0187 (11)	0.0007 (7)	−0.0001 (8)	0.0021 (8)
C17	0.0128 (8)	0.0127 (9)	0.0177 (10)	0.0005 (6)	0.0023 (8)	0.0008 (7)
C18	0.0168 (9)	0.0136 (9)	0.0237 (12)	0.0014 (7)	0.0034 (8)	0.0021 (8)
C19	0.0145 (9)	0.0135 (9)	0.0305 (13)	−0.0027 (7)	0.0049 (9)	−0.0030 (9)
C20	0.0128 (9)	0.0174 (9)	0.0234 (12)	−0.0010 (7)	0.0017 (8)	−0.0036 (8)
S2	0.0176 (2)	0.0167 (2)	0.0169 (2)	−0.00542 (17)	−0.00261 (18)	−0.00039 (18)
N3	0.0128 (8)	0.0136 (8)	0.0203 (10)	−0.0009 (6)	0.0003 (7)	0.0014 (7)
N4	0.0118 (8)	0.0137 (7)	0.0184 (9)	0.0005 (6)	−0.0018 (7)	−0.0013 (7)
C21	0.0126 (8)	0.0121 (8)	0.0152 (10)	−0.0001 (7)	−0.0002 (7)	0.0002 (7)
C22	0.0132 (8)	0.0126 (8)	0.0171 (11)	0.0000 (6)	−0.0024 (8)	0.0009 (8)
C23	0.0160 (9)	0.0118 (8)	0.0185 (11)	0.0003 (7)	−0.0025 (8)	0.0018 (8)
C24	0.0202 (10)	0.0190 (10)	0.0174 (11)	−0.0002 (8)	0.0015 (8)	0.0037 (8)
C25	0.0179 (9)	0.0189 (10)	0.0216 (11)	−0.0048 (7)	−0.0009 (8)	0.0049 (8)
C26	0.0236 (10)	0.0140 (9)	0.0151 (11)	0.0003 (7)	−0.0006 (8)	−0.0006 (8)
C27	0.0204 (9)	0.0126 (9)	0.0171 (11)	−0.0038 (7)	−0.0007 (8)	−0.0002 (8)
C28	0.0201 (10)	0.0192 (10)	0.0197 (12)	−0.0020 (8)	0.0011 (8)	−0.0014 (8)
C29	0.0276 (11)	0.0232 (11)	0.0165 (11)	−0.0065 (9)	0.0008 (9)	−0.0013 (9)
C30	0.0275 (11)	0.0173 (9)	0.0214 (12)	−0.0063 (8)	−0.0076 (9)	0.0036 (9)
C31	0.0240 (11)	0.0145 (9)	0.0268 (13)	−0.0006 (8)	−0.0050 (9)	−0.0007 (8)
C32	0.0246 (10)	0.0159 (9)	0.0181 (11)	−0.0009 (8)	−0.0010 (9)	−0.0032 (8)
C33	0.0150 (9)	0.0123 (8)	0.0149 (10)	−0.0001 (7)	−0.0010 (8)	0.0003 (7)
C34	0.0145 (9)	0.0150 (9)	0.0219 (11)	0.0015 (7)	0.0028 (8)	0.0008 (8)
C35	0.0170 (9)	0.0124 (9)	0.0237 (12)	0.0028 (7)	−0.0013 (9)	−0.0005 (8)
C36	0.0184 (10)	0.0111 (8)	0.0194 (11)	−0.0012 (7)	−0.0012 (8)	0.0018 (8)
C37	0.0138 (9)	0.0126 (8)	0.0157 (10)	−0.0004 (7)	−0.0008 (7)	0.0018 (7)
C38	0.0127 (9)	0.0162 (9)	0.0196 (11)	−0.0005 (7)	−0.0015 (8)	0.0023 (8)
C39	0.0133 (9)	0.0159 (9)	0.0244 (12)	0.0021 (7)	0.0036 (8)	0.0046 (8)
C40	0.0182 (9)	0.0125 (9)	0.0194 (11)	0.0016 (7)	0.0035 (8)	−0.0003 (8)

### Geometric parameters (Å, º)

**Table:**

S1—C5	1.717 (2)	S2—C25	1.718 (2)
S1—C2	1.736 (2)	S2—C22	1.733 (2)
N1—C16	1.368 (3)	N3—C33	1.370 (3)
N1—C13	1.371 (3)	N3—C36	1.373 (3)
N1—H1N	0.87 (3)	N3—H3N	0.87 (3)
N2—C17	1.367 (3)	N4—C40	1.372 (3)
N2—C20	1.374 (3)	N4—C37	1.373 (3)
N2—H2N	0.84 (3)	N4—H4N	0.91 (3)
C1—C17	1.508 (3)	C21—C37	1.506 (3)
C1—C13	1.513 (3)	C21—C33	1.515 (3)
C1—C2	1.516 (3)	C21—C22	1.516 (3)
C1—H1	1.0000	C21—H21	1.0000
C2—C3	1.363 (3)	C22—C23	1.367 (3)
C3—C4	1.433 (3)	C23—C24	1.435 (3)
C3—C6	1.510 (3)	C23—C26	1.511 (3)
C4—C5	1.358 (3)	C24—C25	1.360 (3)
C4—H4	0.9500	C24—H24	0.9500
C5—H5	0.9500	C25—H25	0.9500
C6—C7	1.519 (3)	C26—C27	1.518 (3)
C6—H6A	0.9900	C26—H26A	0.9900
C6—H6B	0.9900	C26—H26B	0.9900
C7—C8	1.388 (3)	C27—C28	1.392 (3)
C7—C12	1.401 (3)	C27—C32	1.397 (3)
C8—C9	1.395 (4)	C28—C29	1.399 (3)
C8—H8	0.9500	C28—H28	0.9500
C9—C10	1.384 (4)	C29—C30	1.386 (3)
C9—H9	0.9500	C29—H29	0.9500
C10—C11	1.389 (4)	C30—C31	1.388 (4)
C10—H10	0.9500	C30—H30	0.9500
C11—C12	1.392 (4)	C31—C32	1.393 (3)
C11—H11	0.9500	C31—H31	0.9500
C12—H12	0.9500	C32—H32	0.9500
C13—C14	1.376 (3)	C33—C34	1.377 (3)
C14—C15	1.424 (3)	C34—C35	1.422 (3)
C14—H14	0.9500	C34—H34	0.9500
C15—C16	1.372 (3)	C35—C36	1.370 (3)
C15—H15	0.9500	C35—H35	0.9500
C16—H16	0.9500	C36—H36	0.9500
C17—C18	1.380 (3)	C37—C38	1.380 (3)
C18—C19	1.419 (3)	C38—C39	1.418 (3)
C18—H18	0.9500	C38—H38	0.9500
C19—C20	1.380 (3)	C39—C40	1.373 (3)
C19—H19	0.9500	C39—H39	0.9500
C20—H20	0.9500	C40—H40	0.9500
			
C5—S1—C2	91.97 (11)	C25—S2—C22	92.07 (11)
C16—N1—C13	109.97 (17)	C33—N3—C36	109.93 (18)
C16—N1—H1N	128 (2)	C33—N3—H3N	120 (2)
C13—N1—H1N	122 (2)	C36—N3—H3N	130 (2)
C17—N2—C20	110.05 (18)	C40—N4—C37	109.73 (18)
C17—N2—H2N	126 (2)	C40—N4—H4N	125.4 (19)
C20—N2—H2N	124 (2)	C37—N4—H4N	124.8 (19)
C17—C1—C13	113.00 (18)	C37—C21—C33	112.67 (18)
C17—C1—C2	114.11 (16)	C37—C21—C22	114.41 (16)
C13—C1—C2	110.00 (16)	C33—C21—C22	110.03 (16)
C17—C1—H1	106.4	C37—C21—H21	106.4
C13—C1—H1	106.4	C33—C21—H21	106.4
C2—C1—H1	106.4	C22—C21—H21	106.4
C3—C2—C1	127.62 (18)	C23—C22—C21	127.31 (18)
C3—C2—S1	111.20 (16)	C23—C22—S2	111.21 (15)
C1—C2—S1	120.97 (17)	C21—C22—S2	121.28 (16)
C2—C3—C4	112.14 (19)	C22—C23—C24	112.16 (19)
C2—C3—C6	125.35 (19)	C22—C23—C26	125.44 (19)
C4—C3—C6	122.5 (2)	C24—C23—C26	122.4 (2)
C5—C4—C3	113.3 (2)	C25—C24—C23	113.1 (2)
C5—C4—H4	123.4	C25—C24—H24	123.5
C3—C4—H4	123.4	C23—C24—H24	123.5
C4—C5—S1	111.42 (17)	C24—C25—S2	111.47 (16)
C4—C5—H5	124.3	C24—C25—H25	124.3
S1—C5—H5	124.3	S2—C25—H25	124.3
C3—C6—C7	112.39 (17)	C23—C26—C27	112.14 (17)
C3—C6—H6A	109.1	C23—C26—H26A	109.2
C7—C6—H6A	109.1	C27—C26—H26A	109.2
C3—C6—H6B	109.1	C23—C26—H26B	109.2
C7—C6—H6B	109.1	C27—C26—H26B	109.2
H6A—C6—H6B	107.9	H26A—C26—H26B	107.9
C8—C7—C12	118.6 (2)	C28—C27—C32	118.7 (2)
C8—C7—C6	121.4 (2)	C28—C27—C26	121.2 (2)
C12—C7—C6	120.0 (2)	C32—C27—C26	120.1 (2)
C7—C8—C9	120.7 (2)	C27—C28—C29	120.6 (2)
C7—C8—H8	119.7	C27—C28—H28	119.7
C9—C8—H8	119.7	C29—C28—H28	119.7
C10—C9—C8	120.5 (2)	C30—C29—C28	120.3 (2)
C10—C9—H9	119.8	C30—C29—H29	119.9
C8—C9—H9	119.8	C28—C29—H29	119.9
C9—C10—C11	119.4 (2)	C29—C30—C31	119.4 (2)
C9—C10—H10	120.3	C29—C30—H30	120.3
C11—C10—H10	120.3	C31—C30—H30	120.3
C10—C11—C12	120.3 (2)	C30—C31—C32	120.5 (2)
C10—C11—H11	119.9	C30—C31—H31	119.8
C12—C11—H11	119.9	C32—C31—H31	119.8
C11—C12—C7	120.6 (2)	C31—C32—C27	120.6 (2)
C11—C12—H12	119.7	C31—C32—H32	119.7
C7—C12—H12	119.7	C27—C32—H32	119.7
N1—C13—C14	107.44 (18)	N3—C33—C34	107.54 (18)
N1—C13—C1	121.91 (17)	N3—C33—C21	121.77 (18)
C14—C13—C1	130.59 (19)	C34—C33—C21	130.56 (19)
C13—C14—C15	107.50 (19)	C33—C34—C35	107.30 (19)
C13—C14—H14	126.3	C33—C34—H34	126.3
C15—C14—H14	126.3	C35—C34—H34	126.3
C16—C15—C14	107.19 (19)	C36—C35—C34	107.60 (19)
C16—C15—H15	126.4	C36—C35—H35	126.2
C14—C15—H15	126.4	C34—C35—H35	126.2
N1—C16—C15	107.90 (19)	C35—C36—N3	107.62 (19)
N1—C16—H16	126.0	C35—C36—H36	126.2
C15—C16—H16	126.0	N3—C36—H36	126.2
N2—C17—C18	107.57 (19)	N4—C37—C38	107.43 (19)
N2—C17—C1	123.30 (17)	N4—C37—C21	123.56 (18)
C18—C17—C1	129.1 (2)	C38—C37—C21	129.0 (2)
C17—C18—C19	107.6 (2)	C37—C38—C39	107.6 (2)
C17—C18—H18	126.2	C37—C38—H38	126.2
C19—C18—H18	126.2	C39—C38—H38	126.2
C20—C19—C18	107.31 (19)	C40—C39—C38	107.39 (19)
C20—C19—H19	126.3	C40—C39—H39	126.3
C18—C19—H19	126.3	C38—C39—H39	126.3
N2—C20—C19	107.5 (2)	N4—C40—C39	107.89 (19)
N2—C20—H20	126.2	N4—C40—H40	126.1
C19—C20—H20	126.2	C39—C40—H40	126.1
			
C17—C1—C2—C3	141.6 (2)	C37—C21—C22—C23	−140.9 (2)
C13—C1—C2—C3	−90.2 (3)	C33—C21—C22—C23	91.1 (2)
C17—C1—C2—S1	−44.1 (2)	C37—C21—C22—S2	44.7 (2)
C13—C1—C2—S1	84.15 (19)	C33—C21—C22—S2	−83.3 (2)
C5—S1—C2—C3	0.81 (17)	C25—S2—C22—C23	−0.63 (17)
C5—S1—C2—C1	−174.41 (17)	C25—S2—C22—C21	174.56 (17)
C1—C2—C3—C4	174.28 (19)	C21—C22—C23—C24	−174.53 (19)
S1—C2—C3—C4	−0.5 (2)	S2—C22—C23—C24	0.3 (2)
C1—C2—C3—C6	−6.8 (3)	C21—C22—C23—C26	6.8 (3)
S1—C2—C3—C6	178.40 (16)	S2—C22—C23—C26	−178.37 (16)
C2—C3—C4—C5	−0.1 (3)	C22—C23—C24—C25	0.3 (3)
C6—C3—C4—C5	−179.1 (2)	C26—C23—C24—C25	179.0 (2)
C3—C4—C5—S1	0.7 (2)	C23—C24—C25—S2	−0.8 (2)
C2—S1—C5—C4	−0.88 (18)	C22—S2—C25—C24	0.80 (18)
C2—C3—C6—C7	−115.1 (2)	C22—C23—C26—C27	114.7 (2)
C4—C3—C6—C7	63.7 (3)	C24—C23—C26—C27	−63.9 (3)
C3—C6—C7—C8	−120.0 (2)	C23—C26—C27—C28	118.9 (2)
C3—C6—C7—C12	60.1 (3)	C23—C26—C27—C32	−60.9 (3)
C12—C7—C8—C9	−0.3 (3)	C32—C27—C28—C29	0.0 (3)
C6—C7—C8—C9	179.8 (2)	C26—C27—C28—C29	−179.7 (2)
C7—C8—C9—C10	0.7 (3)	C27—C28—C29—C30	−0.4 (3)
C8—C9—C10—C11	−0.6 (3)	C28—C29—C30—C31	0.3 (3)
C9—C10—C11—C12	0.1 (3)	C29—C30—C31—C32	0.3 (3)
C10—C11—C12—C7	0.3 (3)	C30—C31—C32—C27	−0.6 (3)
C8—C7—C12—C11	−0.3 (3)	C28—C27—C32—C31	0.5 (3)
C6—C7—C12—C11	179.67 (19)	C26—C27—C32—C31	−179.75 (19)
C16—N1—C13—C14	0.0 (2)	C36—N3—C33—C34	0.6 (2)
C16—N1—C13—C1	−177.46 (19)	C36—N3—C33—C21	176.96 (18)
C17—C1—C13—N1	−47.6 (2)	C37—C21—C33—N3	45.3 (3)
C2—C1—C13—N1	−176.43 (19)	C22—C21—C33—N3	174.30 (19)
C17—C1—C13—C14	135.5 (2)	C37—C21—C33—C34	−139.3 (2)
C2—C1—C13—C14	6.7 (3)	C22—C21—C33—C34	−10.3 (3)
N1—C13—C14—C15	0.0 (2)	N3—C33—C34—C35	−0.4 (3)
C1—C13—C14—C15	177.2 (2)	C21—C33—C34—C35	−176.2 (2)
C13—C14—C15—C16	0.0 (3)	C33—C34—C35—C36	0.0 (3)
C13—N1—C16—C15	0.0 (2)	C34—C35—C36—N3	0.4 (2)
C14—C15—C16—N1	0.0 (3)	C33—N3—C36—C35	−0.7 (2)
C20—N2—C17—C18	0.4 (2)	C40—N4—C37—C38	−0.1 (2)
C20—N2—C17—C1	−177.61 (18)	C40—N4—C37—C21	178.37 (19)
C13—C1—C17—N2	−55.8 (2)	C33—C21—C37—N4	58.7 (3)
C2—C1—C17—N2	70.8 (3)	C22—C21—C37—N4	−68.0 (3)
C13—C1—C17—C18	126.7 (2)	C33—C21—C37—C38	−123.2 (2)
C2—C1—C17—C18	−106.7 (2)	C22—C21—C37—C38	110.2 (2)
N2—C17—C18—C19	−0.3 (2)	N4—C37—C38—C39	−0.1 (2)
C1—C17—C18—C19	177.5 (2)	C21—C37—C38—C39	−178.4 (2)
C17—C18—C19—C20	0.2 (2)	C37—C38—C39—C40	0.2 (2)
C17—N2—C20—C19	−0.3 (2)	C37—N4—C40—C39	0.2 (2)
C18—C19—C20—N2	0.1 (2)	C38—C39—C40—N4	−0.2 (2)

### Hydrogen-bond geometry (Å, º)

*Cg*1–8 are the centroids of the S1/C2–C5, N1/C13–C16, N2/C17–C20, C7–C12, S2/C22–C25, N3/C33–C36, N4/C37–C40 and C27–C32 rings, respectively.

**Table:**

*D*—H···*A*	*D*—H	H···*A*	*D*···*A*	*D*—H···*A*
N1—H1*N*···N3	0.87 (3)	2.61 (3)	3.270 (3)	134 (3)
N4—H4*N*···S2	0.91 (3)	2.86 (3)	3.191 (2)	103 (2)
N1—-H1*N*···*Cg*6	0.87 (3)	2.65 (3)	3.300 (2)	133 (3)
N2—-H2*N*···*Cg*7	0.84 (3)	2.53 (3)	3.249 (2)	145 (3)
N3—-H3*N*···*Cg*3	0.87 (3)	2.70 (3)	3.335 (2)	131 (3)
N4—-H4*N*···*Cg*2	0.91 (3)	2.51 (3)	3.207 (2)	134 (3)
C5—-H5···*Cg*8^i^	0.95	2.98	3.931 (3)	177
C6—-H6*B*···*Cg*8^ii^	0.99	2.79	3.697 (3)	153
C10—-H10···*Cg*7^iii^	0.95	2.86	3.544 (3)	130
C11—-H11···*Cg*5^iii^	0.95	2.98	3.874 (3)	157
C25—-H25···*Cg*4^iv^	0.95	2.98	3.924 (3)	176
C26—-H26*A*···*Cg*4^v^	0.99	2.77	3.684 (3)	153
C30—-H30···*Cg*3^vi^	0.95	2.88	3.585 (3)	132
C31—-H31···*Cg*1^vi^	0.95	2.97	3.863 (3)	156

Symmetry codes: (i) *y*−1, −*x*+2, *z*+1/4; (ii) *y*, −*x*+1, *z*+1/4; (iii) *y*−1, −*x*+1, *z*+1/4; (iv) −*y*+2, *x*+1, *z*−1/4; (v) −*y*+1, *x*, *z*−1/4; (vi) −*y*+1, *x*+1, *z*−1/4.
